# New Specimens of *Yanornis* Indicate a Piscivorous Diet and Modern Alimentary Canal

**DOI:** 10.1371/journal.pone.0095036

**Published:** 2014-04-14

**Authors:** Xiaoting Zheng, Jingmai K. O'Connor, Fritz Huchzermeyer, Xiaoli Wang, Yan Wang, Xiaomei Zhang, Zhonghe Zhou

**Affiliations:** 1 Institute of Geology and Paleontology, Linyi University, Linyi, Shandong, China; 2 Tianyu Natural History Museum of Shandong, Pingyi, Shandong, China; 3 Key Laboratory of Vertebrate Evolution and Human Origin of the Chinese Academy of Sciences, Institute of Vertebrate Paleontology and Paleoanthropology, Beijing, China; 4 Department of Paraclinical Sciences, Faculty of Veterinary Science, University of Pretoria, Onderstepoort, South Africa; Paris Institute of Technology for Life, Food and Environmental Sciences, France

## Abstract

A crop adapted for an herbivorous diet of seeds has previously been documented in the Early Cretaceous birds *Sapeornis* and *Hongshanornis*. Here we report on several specimens of *Yanornis* that preserve a crop containing fish. One specimen preserves two whole fish in the oesophagus, indicating that Early Cretaceous birds shared trophic specializations with Neornithes for the increased energetic demands of flight – namely the storing of food for later consumption when the stomach is full. Whole fish also indicate that despite their presence, teeth were not used to orally process food, suggesting the hypertrophied dentition in this taxon were utilized in prey capture. The presence of macerated fish bones in the crop of other specimens indicates the highly efficient advanced muscular system of peristalsis responsible for moving ingested items between different segments of the alimentary canal was also in place. Despite the fact many features of the modern avian alimentary canal are inferred to compensate for the absence of teeth in birds (expandable oesophagus, grinding gizzard), the derived alimentary canal was apparently present in toothed Cretaceous birds. Although *Yanornis* was considered to have switched their diet from piscivorous to herbivorous, based on position and morphology we reinterpret the gastroliths reported in one specimen as sand impacted in the intestines, and reconstruct the taxon as primarily piscivorous. This is a novel interpretation for fossilized gastroliths, and the first documentation of this condition in the fossil record.

## Introduction

Compared to other groups of vertebrates, living birds have a specialized alimentary canal inferred to have evolved in response to the energetic demands of flight to produce a lightweight and highly efficient digestive system [1], [2]. The Lower Cretaceous Jehol Group has yielded an unprecedented amount of data regarding the biology of early birds. In particular, these fossils have greatly helped to elucidate diet through the preservation of stomach contents, true gizzard stones (geo-gastroliths), crop contents, and potential pellets in numerous specimens that sample the entire avian clade [3]–[7]. These specimens provide clues that allow us to partially reconstruct the alimentary canal of basal birds and start to understand the early evolution of the specialized avian digestive system [7]. The Jehol Group preserves the earliest record of the clade Ornithuromorpha, the lineage which includes living birds – Neornithes [8]. Members of this clade preserve the highest occurrence of stomach contents and other direct dietary clues, suggestive that some of these taxa had already evolved features of the derived alimentary canal that characterize modern birds, including a well-developed, distally located oesophageal crop and a ventriculus (gizzard or muscular stomach) adapted for grinding hard food items [7]. *Yanornis martini* [9] was the first Jehol taxon discovered with preserved evidence of its diet; a referred specimen (IVPP V13259) preserves fish in the abdominal region, interpreted as gut contents [4]. Soon after, another referred specimen (IVPP V13558) was reported preserving a large mass of gastroliths, suggesting the diet of this taxon varied seasonally, similar to many living birds [6]. However, this taxon is also regarded as herbivorous [10]. Here we report on ten new specimens of *Yanornis* sp. that preserve fish remains in the crop as well as the ventriculus and discuss the implications of this discovery on interpretations of the diet of this taxon and hypotheses regarding evolution of the crop and alimentary canal in early avian history.

## Materials and Methods

**Figure 1:**
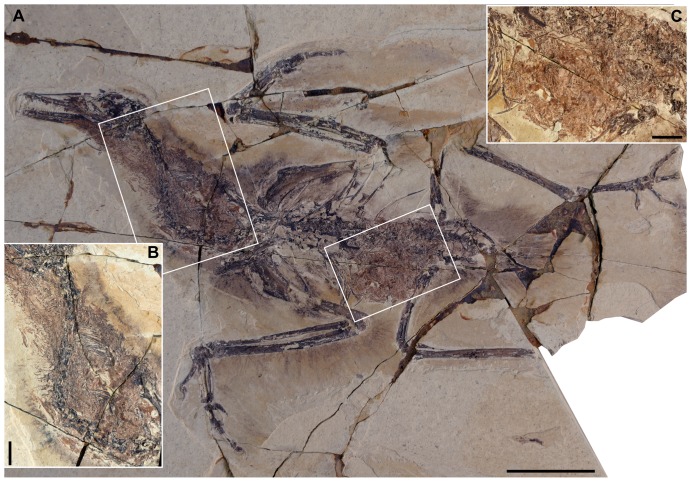
*Yanornis* STM9-15 preserving whole fish in the crop and macerated fish bones in the ventriculus: (A) full slab, scale bar equals five cm; (B) detail of the crop, scale bar equals one cm; (C) detail of the ventriculus, scale bar equals one mm.

**Figure 2:**
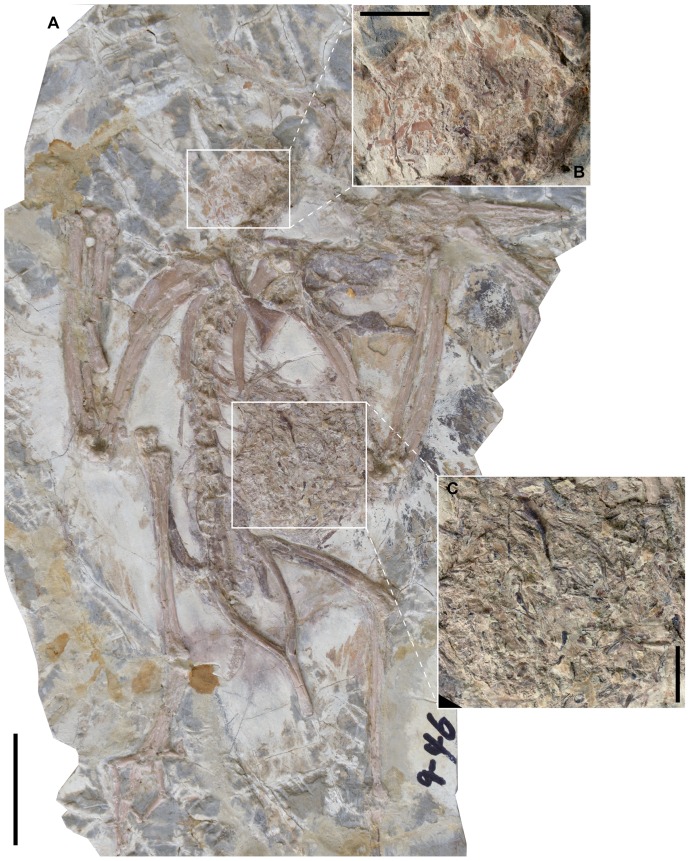
*Yanornis* STM9-46 preserving macerated fish bones in the crop and ventriculus: (A) full slab, scale bar equals four cm; (B) detail of the crop, scale bar equals one cm; (C) detail of the ventriculus, scale bar equals one mm.

**Figure 3:**
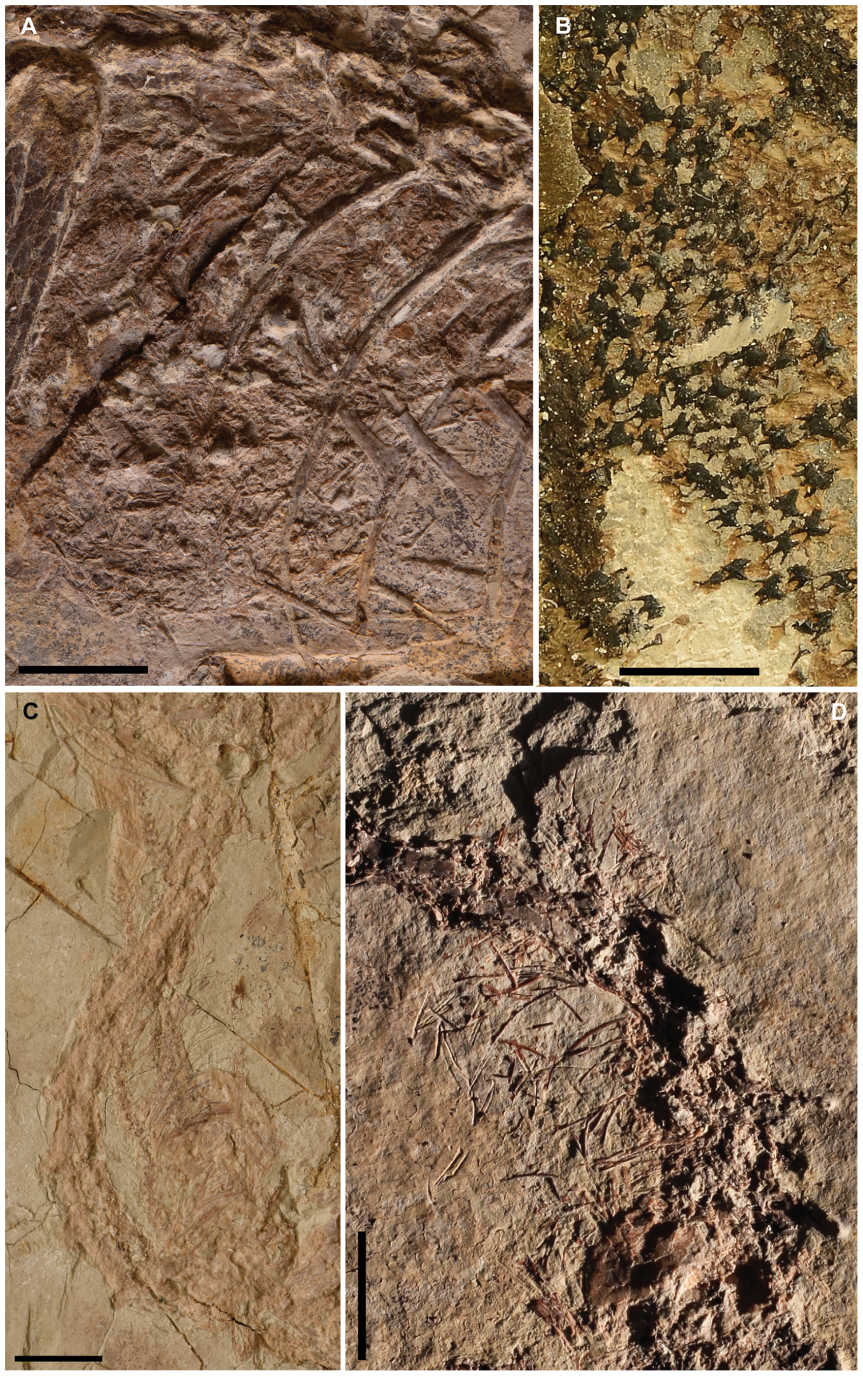
Detail photographs of *Yanornis* specimens preserving fish remains in the alimentary canal: (A) macerated fish bones and a few accidentally ingested stones preserved in the ventriculus of STM9-51; (B) the articulated “microtenoid” scales of the fish *Protopspherus* preserved in the crop of STM9-19; (C) an entire fish, referable to *Jinanichthys*, preserved in the crop of STM9-31; (D) macerated fish bones preserved in the crop of SMT9-18.

A large number of purchased specimens referred to *Yanornis* sp. were examined at the Shandong Tianyu Museum of Nature (STM, Pingyi, Shandong, China). These specimens were purchased by the Director for the Museum collection. We have full permission from the Director (who is lead author and initiated the project) to study this material. No permits were required for the described study, which is based entirely on museum specimens; this research complies with all relevant regulations. The specimens were identified as *Yanornis* based on their proportionately elongate rostrum (55–60% skull length), heavily toothed upper and lower jaws with approximately twenty closely spaced, recurved dentary teeth, and an intermembral index of approximately 1.1 (Table S1 in File S1). Ten specimens were identified preserving fish in the body (Table 1). All ten specimens preserve fish remains in the crop; most of the new specimens also preserve fish remains in the abdominal cavity, and one of these additionally preserves a few gastroliths together with the fish remains (STM9-51; Table 1). All specimens are preserved in articulation and nearly complete (Figs. 1–2, Figs. S1–S10 in File S1). The masses of macerated fish bones in the ventriculus can be clearly identified as in the body based on their clumped association and the fact they are overlapped by ribs in lateral view (Figs. 1, 2, 3A). The fish bones interpreted as in the crop are so inferred based on their position and close association with the neck; in some cases the outline of the body is defined by feather impressions, and the fish bones are clearly located inside the body (Fig. 1, Fig. S2 in File S1). The disarticulated masses of fish bones present in some specimens would also be unlikely to occur unless they had been ingested and macerated in the ventriculus and resemble aggregates of fish bones found in the pellets of modern piscivorous birds (Figs. 2, 3D, Fig. S2 in File S1).

**Table 1:** List of *Yanornis* sp. specimens preserving direct evidence of diet.

Specimen no.	Crop contents	Stomach contents (ventriculus)
STM9-15	>1 whole fish	Disarticulated fish bones
STM9-18	Whole fish, disarticulated fish bones	Disarticulated fish bones
STM9-19	≥1 whole fish	Disarticulated fish bones
STM9-26	≥1 whole fish, soft tissue	Preservation unclear
STM9-31	1 whole fish	Disarticulated fish bones
STM9-37	Fish bones	None
STM9-46	Disarticulated fish bones	Disarticulated fish bones
STM9-49	1 whole fish	Disarticulated fish bones (fine)
STM9-51	Disarticulated fish bones	Disarticulated fish bones and gastroliths
STM9-52	Disarticulated fish bones	Disarticulated fish bones
IVPP V13358	None	Gastroliths (large intestine)
IVPP V13259	None	Disarticulated fish bones

The expanded portion of the oesophagus or crop, as can be inferred from the position of fish bones ventral to the cervical vertebrae, extends from the third to the 11^th^ cervical vertebra (Figs. 1A,B, 3C,D, Figs. S1, S3, S5 in File S1). Its size may be exaggerated by compression of the full, three-dimensional structure (Fig. S1 in File S1). As in living birds, when the head is preserved erect and the neck is in the S-shaped position, the crop extends ventral to the cervical vertebrae proximally and dorsal to them distally (Figs. 1, 3C). Several specimens preserve what appear to be the remains of whole fish (Figs. S1, S4, S5, S8 in File S1); in one specimen (STM9-19; Fig. 3B, Fig. S1 in File S1), a large patch of “microctenoid” fish scales are preserved in articulation, referable to the basal paddlefish *Protopspherus* sp. [11]. Three specimens (STM9-15, STM9-26, STM9-49; Fig. 1, Figs. S4, S5 in File S1) preserve nearly complete skeletons of articulated fish bones, tentatively identified as *Jinanichthys*, a basal osteoglossomorph common in the Jehol [12]. STM9-15 preserves at least two complete fish, which we estimate would have measured 70–80 mm in body length (Fig. 2). In other specimens (STM9-46, -51, -52; Figs. 1, 3D, Figs. S9, S10 in File S1) the fish bones are disarticulated, interpreted as macerated, hard to digest fish remains egested from the stomachs, ready to be regurgitated as a pellet [13].

The macerated fish remains in the abdominal cavity are located just cranioventral to the pelvic girdle and are interpreted as in the gizzard (Figs. S6, S7, S9, S10 in File S1). The location of the macerated fish remains is consistent in all specimens and more caudally located than the inferred position of the proventriculus (glandular stomach). One specimen (STM9-51; Fig. S9 in File S1) preserves three to five gastroliths (1.1–1.9 mm) dispersed throughout the macerated fish remains (Fig. 3A). The consistent position and consolidated nature of the fish bones, in either the crop or ventriculus, suggests that the macerated fish bones were not displaced into the oesophagus post-mortem (no fish bones preserved in the proventriculus, where digesta has a relatively short residency time [2]).

## Discussion

### Dietary inferences regarding *Yanornis*

**Figure 4:**
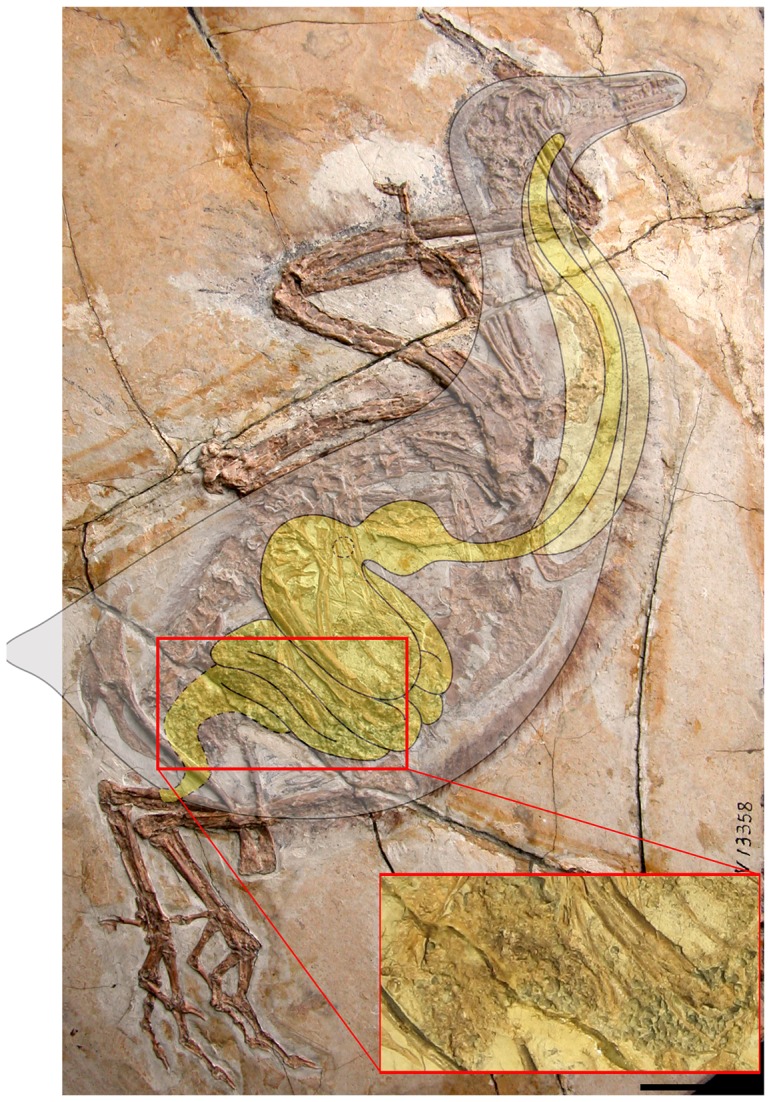
Interpretative drawing of the alimentary canal of *Yanornis* superimposed over IVPP V13558. Enlarged red box shows preservation of sand compacted in the intestines. The grit is clearly too caudally located to represent gizzard stones. The oesophagus is drawn in a way to demonstrate its flexibility; it leads to the small proventriculus (pyloric sphincter indicated by dashed circle), followed by the larger ventriculus, and then the intestines and cloaca. Scale bar equals 1 cm.

Until now, the diet of *Yanornis* has been ambiguous; early reports considered the taxon piscivorous based on the association of fish remains with one specimen [4]. Extant piscivorous birds do not utilize gastroliths – they are not necessary to break down this soft food; when a specimen of *Yanornis* was reported preserving a large mass of gastroliths (IVPP V13358), the taxon was interpreted as having seasonal changes in diet, spending part of the year as an herbivore [6]. However, perhaps because *Yanornis* was the first Mesozoic bird to preserve gastroliths, this taxon has been largely considered herbivorous [10]. Within Theropoda there are morphological characters that are correlated with herbivory – several of these are related to the loss of teeth [10]. The basal ornithuromorph *Archaeorhynchus* is edentulous, possessing several morphological indicators of herbivory, and also preserves geo-gastrolith gizzard stones in all specimens [3], [14], thus direct evidence and morphological indicators concur. However, most *Yanornis* specimens preserve fish remains and do not preserve gastroliths, although two specimens do preserve the latter feature (IVPP V13558, STM9-51). Furthermore, morphological indicators for herbivory are absent; *Yanornis* possesses the most robust, sectorial dentition of any Early Cretaceous ornithuromorph and a greater number of teeth than any Early Cretaceous bird. Furthermore, in light of new comparative material, the gastroliths preserved in *Yanornis* show some peculiarities. In specimens of *Archaeorhynchus* the gizzard stones are all similar in size (2 mm), whereas in IVPP V13558 the stones range from 0.2 – 2.7 mm, and a large number of them are smaller than the gizzard stones preserved in *Hongshanornis* (∼1 mm), a much smaller taxon [7]. The stones are also far more numerous in IVPP V13558. While nearly every other specimen with gastroliths is preserved in dorsoventral view, showing a small, dense, round mass of stones just distal to the sternum, *Yanornis* IVPP V13558 is preserved in lateral view; the entire specimen is in articulation and preserves the outline of the body (Fig. 4). The stones are more caudoventrally located than in *Archaeorhynchus*, and extend dorsoventrally through the entire body cavity, occupying a much greater area than preserved masses of gizzard stones (*Archaeorhynchus*, *Hongshanornis*). This position is more consistent with the intestines than the gizzard (Fig. 4). The smaller size, greater number and size range, and more caudal location of the stones, together with the large number of specimens preserving fish remains described here, suggest an alternate interpretation of the stones in IVPP V13558: that they represent sand impaction in the intestinal tract. In such a case, an intestinal blockage (such as plant material or feathers accidentally ingested during feeding, or pathological growths within the intestinal tract) prevents stones accidentally ingested during feeding from passing through the body [15]. In extant birds, a few grains of sand are ingested [16], particularly when eating dead fish left on land by receding water. These grains of sand are carried along with the intestinal contents, which are liquid in the small intestine, until they reach the large intestine, where water is re-absorbed from the intestinal contents. Here the sand settles in a ventral-most loop, where it remains and slowly accumulates until the intestine is blocked, eventually causing death. In IVPP V13558 the mass of gastroliths form distinct lobes that extend dorsoventrally through the body, revealing the first fossilized glimpse of the intestines of a Mesozoic bird (Fig. 4). Depending on the rate of accidental ingestion, impaction can occur slowly or quickly (F. Huchzermeyer pers. obs.). Approximately 1% of shorebirds suffer this fate, which can also occur in gallinaceous birds (F. Huchzermeyer pers. obs.) [17] and captive ratites [15]; the condition is normally rare (although there are numerous recent reports of impaction due to ingestion of man-made waste [18]), but it is not inconceivable that this ailment was captured by the fossil record. The three to five gastroliths preserved in the gizzard of *Yanornis* STM9-51 (Fig. 3A) are consistent with the accidental ingestion of a small number of stones while feeding that would normally pass through the digestive tract or be regurgitated [16], and thus they are not true gizzard stones. In *Yanornis* IVPP V13558, such stones and other sand particles accidentally ingested were unable to pass through the large intestine due to an obstruction and likely caused the death of this individual. Living osteoglossomorphs, distant relatives of *Jinanichthys*, are mostly pelagic or benthopelagic and it would be unlikely that *Yanornis* would ingest stones catching fish in open water. However, the large amounts of volcanic activity during the deposition of the Jehol probably would have disrupted the pH of the lake, resulting in mass fish mortalities that would have provided abundant food available for scavenging on the shore. These small stones were most likely ingested while *Yanornis* was opportunistically foraging on the lakeshore, consistent with extant shorebirds that occasionally suffer from sand impaction.

### Evolution of the avian crop

The oesophagus of living birds is a specialized muscular organ, proportionately larger and wider than in other groups of vertebrates [1], [2]. This is inferred to allow birds to swallow large food items with minimal oral processing in the absence of teeth [1], [19]. The crop is an expansion of the oesophagus to which food is diverted when the proventriculus is full, thus allowing birds to collect more food than they are capable of immediately digesting [2]. This is important for birds because flight is energetically demanding and requires a large amount of food to sustain it [2]. The morphology of the crop ranges from a simple swelling of the midsection of the oesophagus in some taxa (e.g. piscivorous birds), to forming well developed distinct, distally located lobes in others (e.g. gallinaceous birds, water fowl, Columbiformes). A well-developed crop is most commonly present in granivorous birds whose ventriculus has been specialized for grinding hard seeds and thus can no longer be used to simply contain large amounts of food [2]. Most but not all birds possess a crop [2]. Modern birds have adapted to a huge diversity of trophic niches and depending on the diet, a crop may not be efficient; some food sources, such as fruit, spoil too quickly to keep inside the body. During the course of avian evolution, the crop has both been lost as well as become highly specialized. Most birds of prey possess a crop with the notable exception of owls, which have a specialized digestive tract and can only process one meal at a time, not eating again until the previous meal's pellet has been regurgitated, thus obviating the need for this structure [20], [21]. The South American stink bird, or Hoatzin (*Opisthocomus hoazin*), has a specialized enlarged crop where vegetative matter is kept for a period of days (giving this taxon its colloquial name) before it passes on to the stomach [2]; this crop is homologous to the mammalian rumen and an example of evolutionary convergence [22]. The size of the crop in this bird has resulted in skeletal modifications to contain the enlarged organ. The neornithine crop has multiple functions related to the derived avian lifestyle; it is also used by some altricial birds to soften and store food to later be egested back at the nest to feed their young [2]. It is even specialized to produce ‘crop milk’ in Columbiformes (and also flamingos and some penguins), which is fed to young [2], and regulated by the same hormone that produces milk in mammals [23].

A crop full of seeds has been reported in the basal pygostylian *Sapeornis* and the ornithuromorph *Hongshanornis* [7], both also from the Early Cretaceous Jehol Group. Here we document the presence of a crop containing fish in the ornithuromorph *Yanornis*. In living birds, the simplest crops are associated with piscivorous birds (e.g. cormorants, gulls). In these taxa, the crop is only a simple swelling of the middle portion of the oesophagus [2]. In *Yanornis*, the crop appears to extend throughout the neck, from the third to 11^th^ cervical vertebrae, and is located ventral to the cervicals proximally and dorsal to the vertebrae distally, consistent with living taxa with similarly simple crop morphologies [19]. In granivorous birds the crop is more strongly developed, being expanded into a ventrally located diverticulum with a distinct lobed-shape that varies between groups and taxa (one vs. two lobes) [2]. Although the exact shape of the crop cannot be determined due to preservation, the position of the crop in two specimens of *Sapeornis* and one specimen of *Hongshanornis* (as inferred by the position of the preserved seeds) are all more ventrally located than the crop in *Yanornis*, being located ventral to the distal cervicals in *Sapeornis* and near the cervicothoracic transition in *Hongshanornis* [7]. Thus, correlations between diet, crop morphology, and crop position are consistent in living and basal birds: crops associated with soft foods like fish extend throughout the oesophagus and are morphologically simple, whereas crops associated with seeds are distally located and form a distinct ventral expansion.

*Yanornis* STM9-15 preserves more than one whole fish in the crop, indicating that basal ornithuromorphs possessed a flexible oesophagus and also collected more food than could be immediately processed, advantageous for limiting foraging time (in which the bird is often exposed and vulnerable) and meeting the metabolic demands of flight [2]. The presence of disarticulated bones in the crop of several other specimens (Table 1) suggests *Yanornis* regurgitated hard to digest items, as do many groups of living birds (e.g. raptors, cormorants, owls) [2], [13], [24], [25]. Peristalsis, or muscle contractions, of the oesophagus are responsible for moving food from the crop into the proventriculus. Similarly, peristalsis allows food from the ventriculus to be moved back into the proventriculus for further digestion, or to allow indigestible remains to be regurgitated back up through the crop. This further increases the efficiency of the digestive system by reducing the time required to process ingested items as well as the overall weight of the bird as it digests its food [2]. The fact that *Yanornis* is documented with macerated fish bones in both the ventriculus and crop suggests that this taxon had the same complex but highly efficient muscular digestive system as living birds, designed to meet the metabolic demands of powered flight within the physical constraints of aerial locomotion.

Two crop morphologies are now documented among basal ornithuromorphs, a simple mid-oesophageal expansion (*Yanornis*) and a well-developed distal oesophageal pouch (*Hongshanornis*) [7]. This suggests that the crop is plesiomorphic to Ornithuromorpha, and thus potentially Ornithothoraces (the clade formed by Enantiornithes and Ornithuromorpha) although there is no evidence of this feature in Enantiornithes. A derived ventrally located crop is also present in the basal pygostylian *Sapeornis* [7] and the long boney-tailed bird *Jeholornis* is inferred to have a well developed crop from the association of undigested seeds in one specimen [5] (unfortunately, the specimen is disarticulated and the morphology of the crop cannot be determined). Among theropod dinosaurs there is no evidence of a crop outside Aves (although a mummified specimen of *Brachylophosaurus* suggests this lineage of ornithischian dinosaurs may have evolved this feature convergently [26]). The presence of gastroliths preserved in the abdominal cavity of several specimens of *Caudipteryx* (Maniraptora: Oviraptorosauria) suggests that the derived two-part stomach evolved outside Aves [27]. Cladistic analysis of Aves (not including *Caudipteryx*) indicates that a grinding gizzard was plesiomorphic to the Sapeornithiformes + Ornithothoraces clade, and secondarily lost in Enantiornithes (see Supplementary Information). The presence of a crop is resolved as a synapomorphy of *Jeholornis* and all more derived birds (Fig. S11 in File S1). We suggest that the original function of the crop was to store food [2] and only in Aves did limitations in body size and the rigid abdomen necessitate the evolution of additional food storage outside the confines of the body cavity.

### Evolution of the avian feeding mechanism

The energetic demands and physical constraints of powered flight in Aves have produced in a number of major modifications that biologically set this group apart from other living vertebrates, and the feeding mechanism (feeding apparatus and alimentary canal) is no exception [1]. The feeding apparatus in Neornithes is characterized by the absence of teeth and reduced jaw muscular while the alimentary canal itself is highly efficient and lightweight, being proportionately shorter than in mammals [28], [29] with reduced residence times for ingested items [1], [2], [30]. The highly efficient alimentary canal is characterized by a number of unique features including the crop and a two-part stomach (both features identified in basal birds), while the intestines show less modification [1]. Although modifications of the feeding apparatus and alimentary canal are inferred to be flight related, only recently is there significant data from the fossil record available to test ornithological hypotheses regarding the evolution of the modern avian digestive system.

The wide and elastic oesophagus that characterizes Neornithes is inferred to have evolved to allow birds to swallow large food items with minimal oral processing in the absence of teeth [1], [19]. The presence of two whole fish in the crop of several specimens of the toothed bird *Yanornis* indicates the crop was highly elastic, thus the evolution of oesophageal elasticity preceded the loss of teeth themselves. Modern piscivorous birds and birds of prey, having no teeth, primarily swallow their prey whole, their flexible oesophagus expanding to accommodate food items [2], [19]. They utilize strong gastric acids to break down their food and regurgitate harder to digest items in a pellet – gizzard stones are not utilized to break down this relatively soft food source [2]. Notably, the presence of whole fish preserved in the crop of several specimens indicates that *Yanornis* did not utilize its numerous large and recurved teeth to process its prey prior to ingestion, but swallowed fish whole with no oral processing as in living carnivorous birds. Compared to other Early Cretaceous birds, teeth are hypertrophied in *Yanornis*; evidence that the teeth were not utilized to process food in turn suggests teeth may have instead been important for prey capture in this species.

Tooth loss in modern birds is inferred to be an adaptation for flight, reducing the overall weight of the skeleton [1]. There is also an inferred correlation between tooth reduction (and eventual loss) and the evolution of the grinding gizzard [7], which functions to break down food in the absence of oral processing and thus obviates the former [1], [2], [7]. Furthermore, in living birds, a grinding gizzard necessitates a large crop, suggesting these two features should also be correlated. Mesozoic birds show the same relationship between crop and gizzard morphology as living birds, namely a large distally located crop is found in specimens also preserving gizzard stones indicative of a well developed ventriculus (*Sapeornis*, *Hongshanornis*), whereas a simple crop is found in taxa lacking gizzard stones (*Yanornis*). Indeed, teeth are reduced in *Hongshanornis* and *Sapeornis*, but not absent [31], [32], and thus grinding gizzards are correlated with tooth reduction. *Yanornis* neither required (based on diet) nor had a grinding gizzard and its teeth are hypertrophied, thus natural selection on the teeth must have been driven by another function, which we have suggested may be prey capture. The combination of crop and gizzard morphologies recognized among basal ornithuromorphs indicates that different lineages had evolved diet specific morphologies of the alimentary canal, very similar to living birds [2]. There is a no clear trend in tooth reduction in basal birds; *Jeholornis* and *Sapeornis* have reduced dentition and *Confuciusornis* is edentulous (but shows no evidence of a grinding gizzard) but most Early Cretaceous enantiornithines and *Yanornis* show the opposite trend, with dentition that is more prominent than the plesiomorphic avian condition. Differences in diet may explain the absence of any clear pattern of tooth loss among basal birds – teeth were not immediately lost in more carnivorous birds such as *Yanornis*, which could still utilize this feature for predation. The persistence of teeth within Aves for the first 100 million years of their evolution (present in the Late Cretaceous *Hesperornis* and *Ichthyornis*) [33] clearly indicates that powered flight did not limit dentition. It is more likely that redundancy with the gizzard, and the more efficient and flexible nature of the latter structure, lead to the eventual loss of teeth within Aves.

## Conclusions

The new specimens described here along with reinterpretations of a previously described specimen indicate that *Yanornis* was a piscivorous bird. The new specimens indicate that *Yanornis* had a simple crop capable of expanding to carry considerable loads, similar to living piscivorous birds. The presence of a simple crop in *Yanornis* suggests this feature was far more widespread within Aves than previously inferred. The presence of macerated fish remains in the crop indicates that the peristalsis mechanism that allows food to move between the crop and proventriculus and the ventriculus and proventriculus was also present, which suggests that *Yanornis* had the same complex but highly efficient muscular digestive system as living birds. Whole fish in the crop indicate that despite the presence of hypertrophied dentition, food was not mechanically processed prior to consumption, suggesting the large recurved teeth in *Yanornis* were used in prey acquisition. This data indicates that the modern avian alimentary canal evolved prior to the modern derived feeding apparatus.

## Supporting Information

File S1 **Combined pdf.** Comprised by Table S1, additional specimen photos Figures S1–10, and the data and results (Fig. S11) of the cladistic analysis.
(PDF)
