# Supplementary material for: New Specimens of Yanornis Indicate a Piscivorous Diet and Modern Alimentary Canal
Source: PLoS One. 2014 Apr 14;9(4):e95036. doi: 10.1371/journal.pone.0095036 (PMC3986254; doi:10.1371/journal.pone.0095036)
Supplement: File S1 — Combined pdf. Comprised by Table S1, additional specimen photos Figures S1–10, and the data and results (Fig. S11) of the cladistic analysis. (PDF) [file pone.0095036.s001.pdf]

Supplementary Information – Zheng et al., *Yanornis* crop

	Skull Length	Coracoid	Scapula	Humerus	Ulna	Carpometacarpus	Femur	Tibiotarsus	Tarsometatarsus
STM9-15	72.2	39	57.8*	80	85	34*	51*	78*	39
STM9-18	60	35	50*	73	79	35	49	70*	31*
STM9-19	62*	_	_	76	80	34.5	58	73	38
STM9-26	72	_	_	78*	80*	_	_	_	_
STM9-31	60	_	_	_	_	_	_	_	52*
STM9-37	68	_	45*	79	86	38*	55*	74	_
STM9-46	68*	34	50	80	86	39*	57	74	40
STM9-49	61*	_	_	72	80*	_	50*	75*	_
STM9-51	64*	30*	42	67	71*	_	57	75	35
STM9-52	68	31	48	68*	72	41	50	68	35

Table S1. Comparative measurements (mm) of the ten referred specimens of *Yanornis* from the STM. The asterisk denotes estimated lengths. Incomplete elements were excluded.

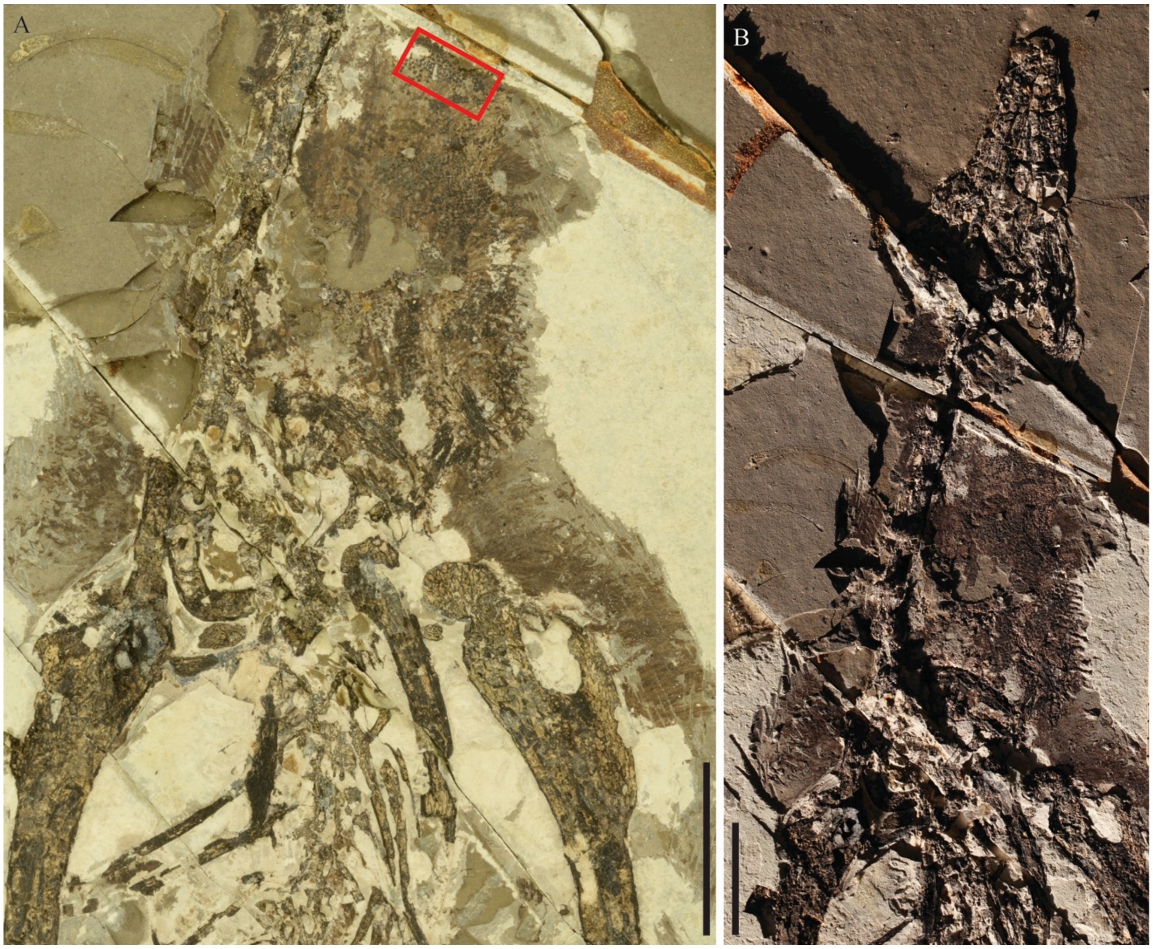

Figure S1. *Yanornis* STM9-19 preserving articulated *Protopspherus* sp. fish scales in the crop: (A), main slab in ventral view; (B) counterslab in dorsal view (skull in ventral view). Area in red box enlarged in Fig. 3B. Scale bars equal two cm.

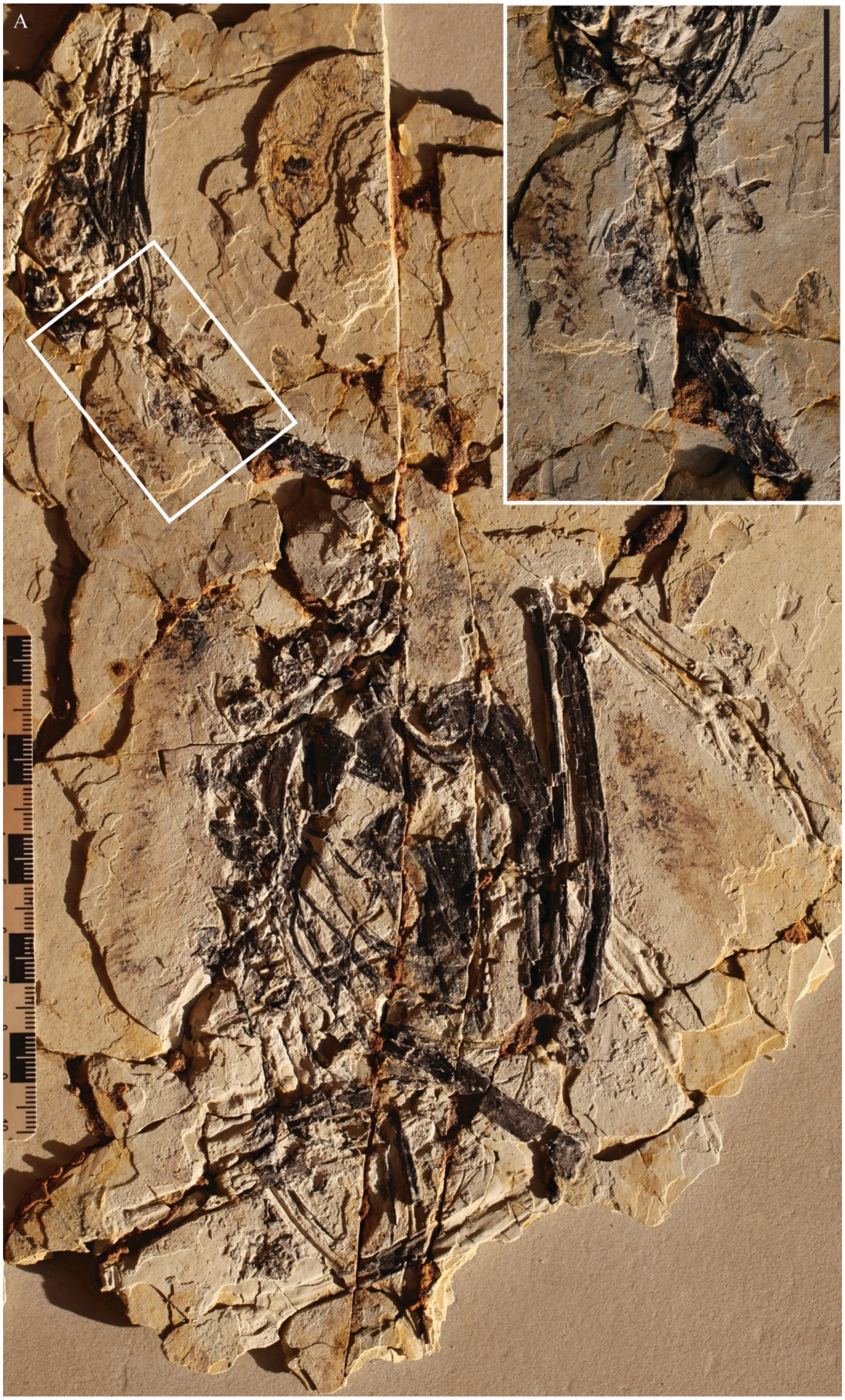

Figure S2. *Yanornis* STM9-37 preserving a small patch of disarticulated fish bones in the crop: (A), full slab, preserved in right lateral view; (B) close up of the fish bones in the crop. Scale bar equal two cm.

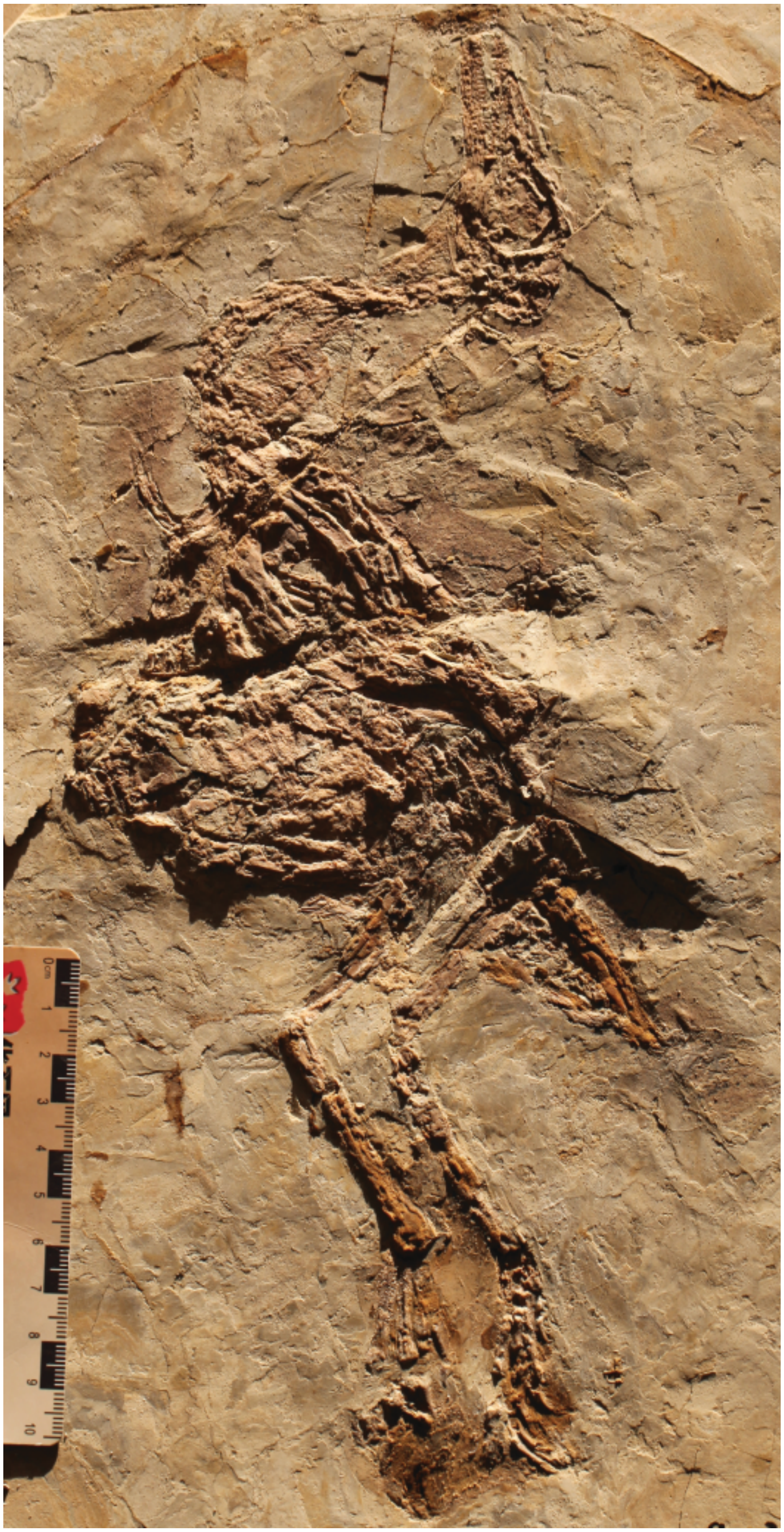

Figure S3. Full slab of *Yanornis* STM9-31 in left lateral view preserving a whole fish in the crop (close up in Fig. 3C) and macerated fish bones in the ventriculus.

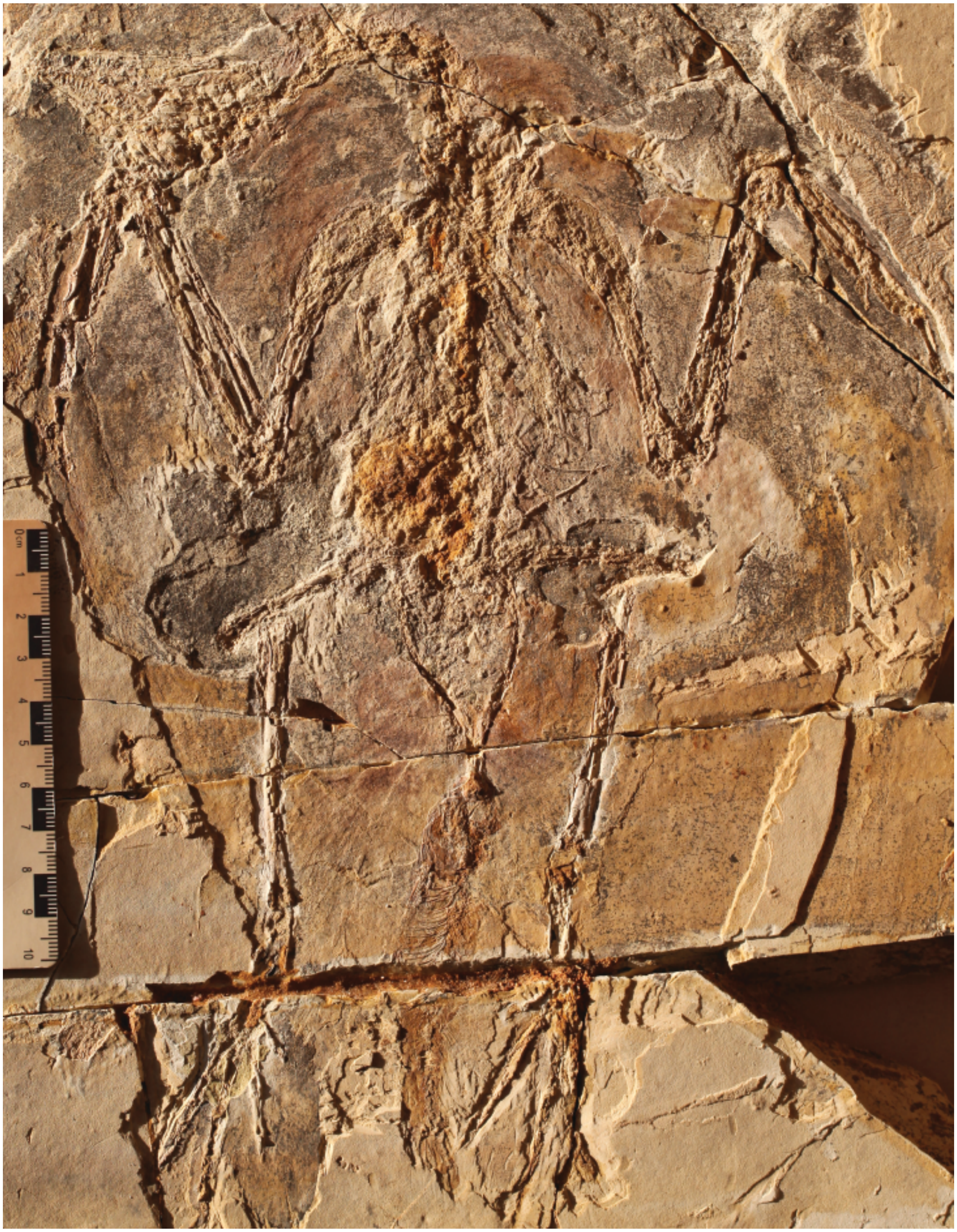

Figure S4. Full slab of *Yanornis* STM9-49 in ventral view preserving a small articulated fish in the crop and macerated fish bones in the ventriculus.

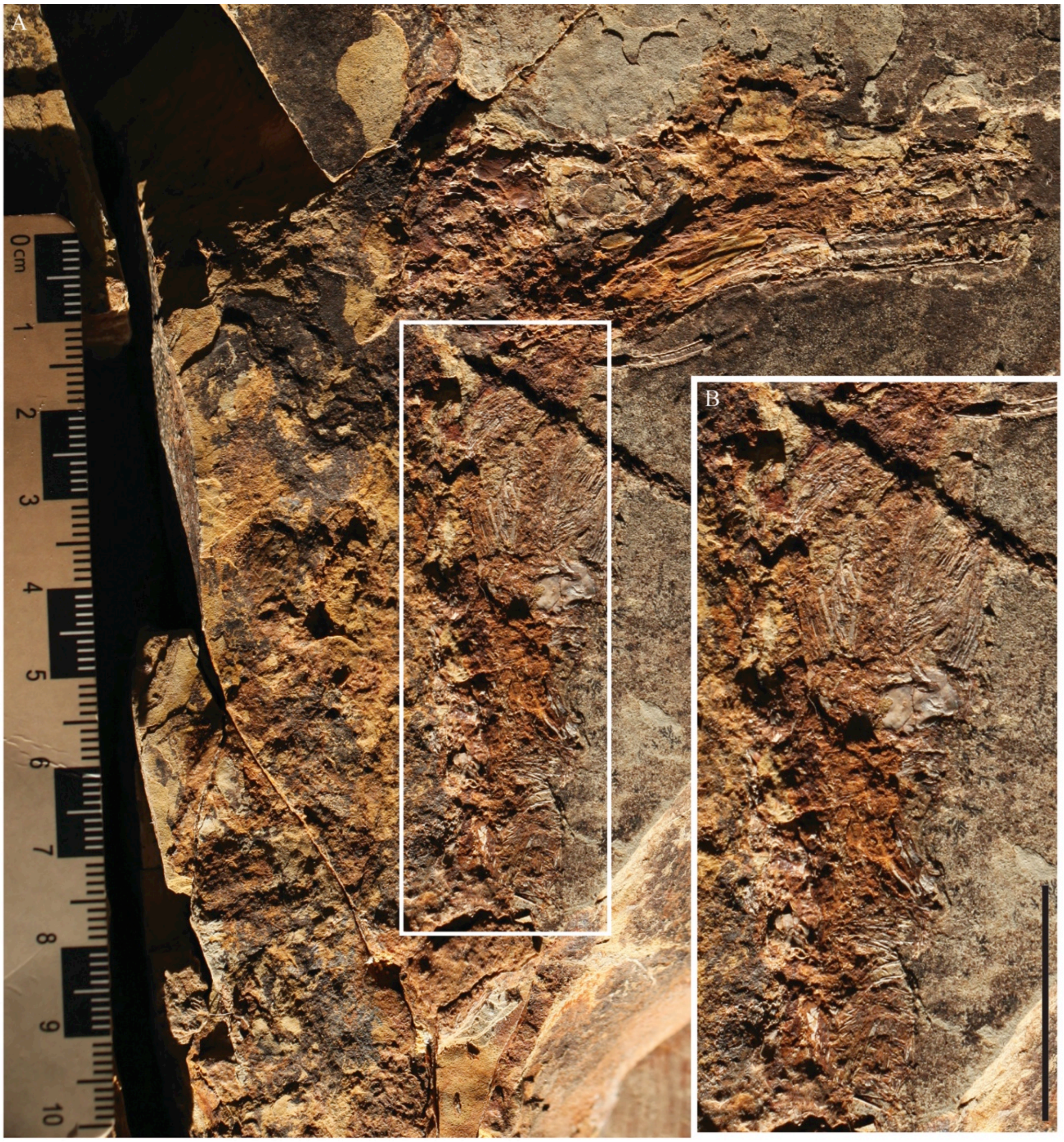

Figure S5. *Yanornis* STM9-26 preserving fish in the crop: (A) head and neck region; (B) close up of articulated fish in crop (area in white square in part A), scale bar equals two cm.

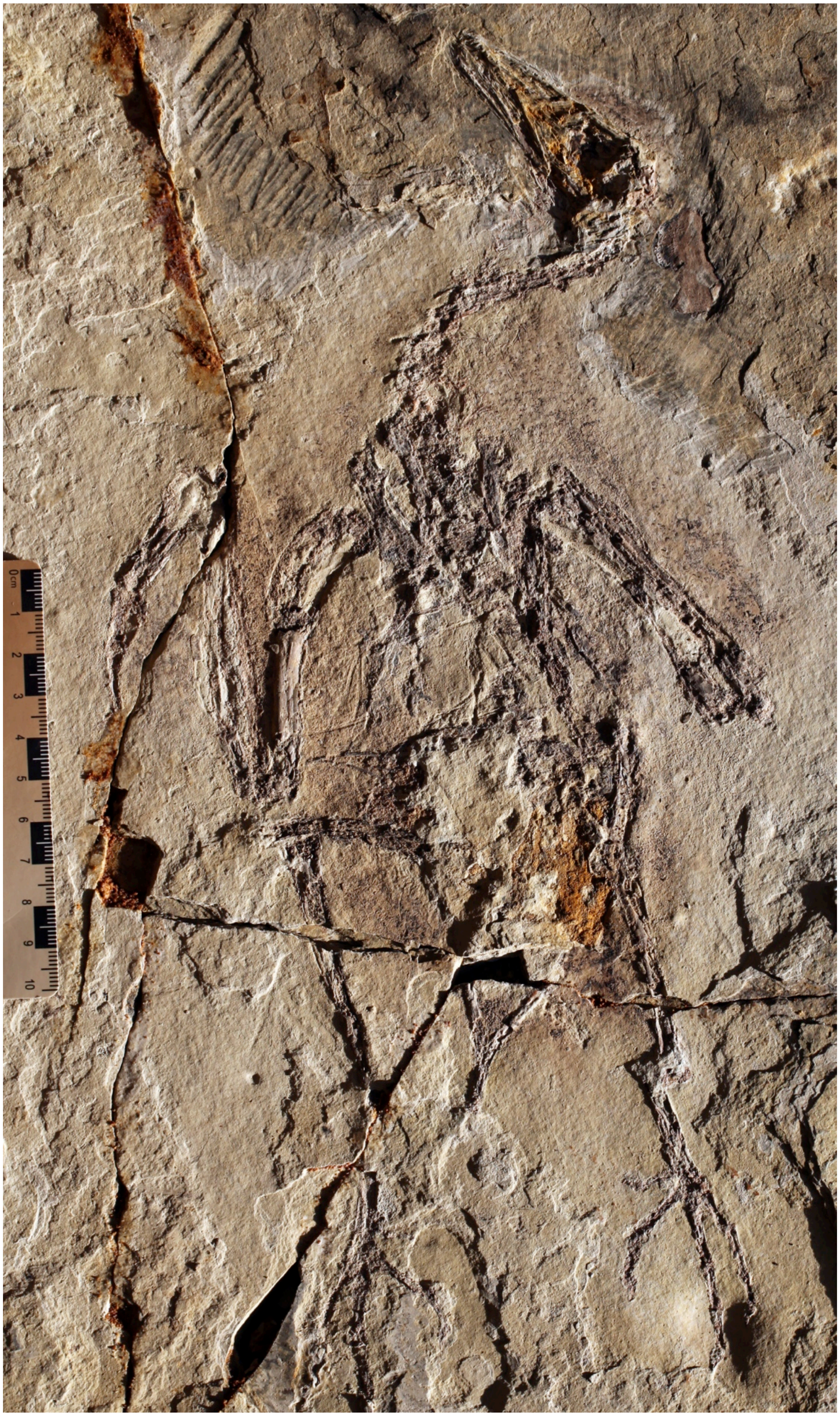

Figure S6. Photograph of *Yanornis* STM9-18

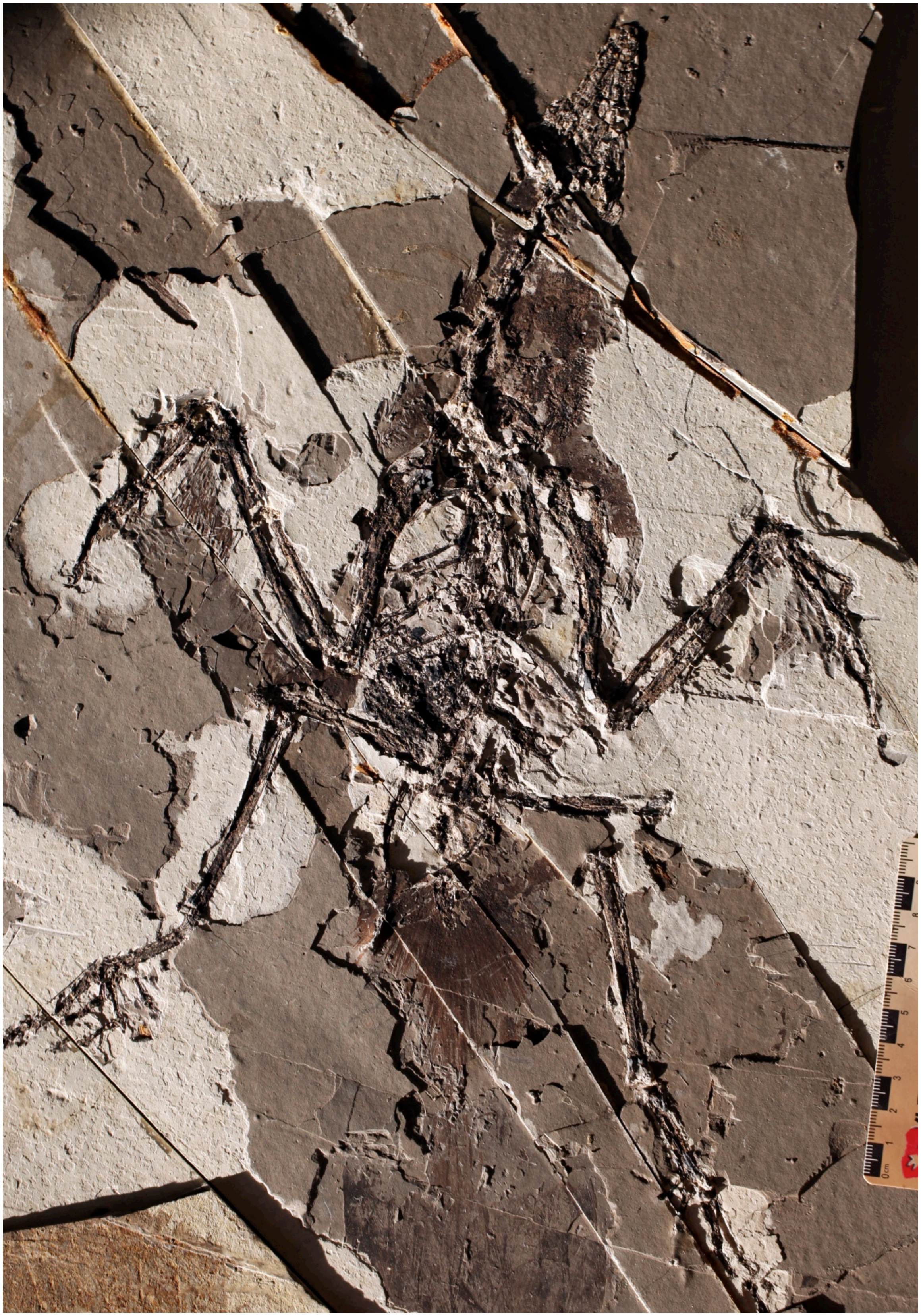

Figure S7. Photograph of *Yanornis* STM9-19

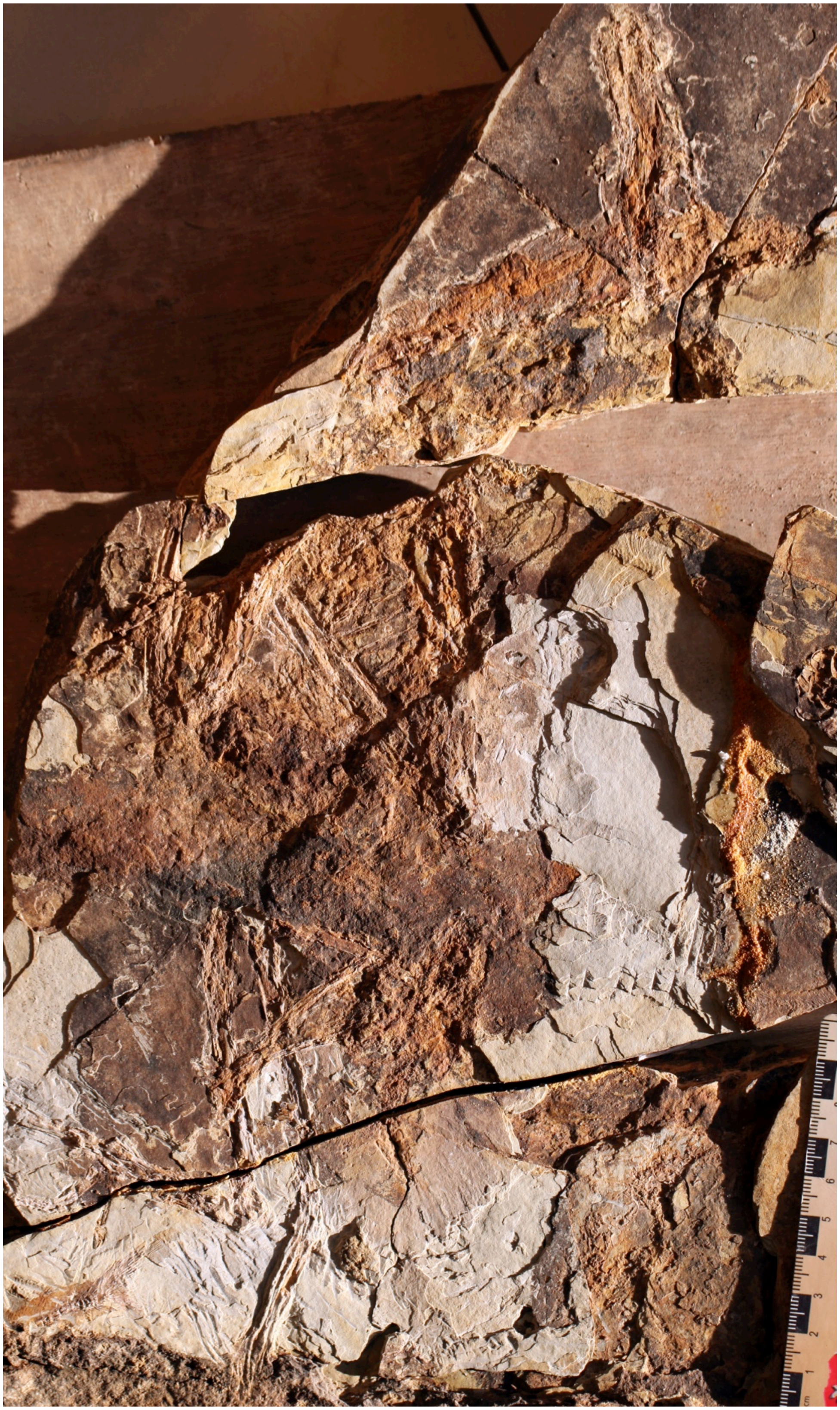

Figure S8. Photograph of *Yanornis* STM9-26, full slab.

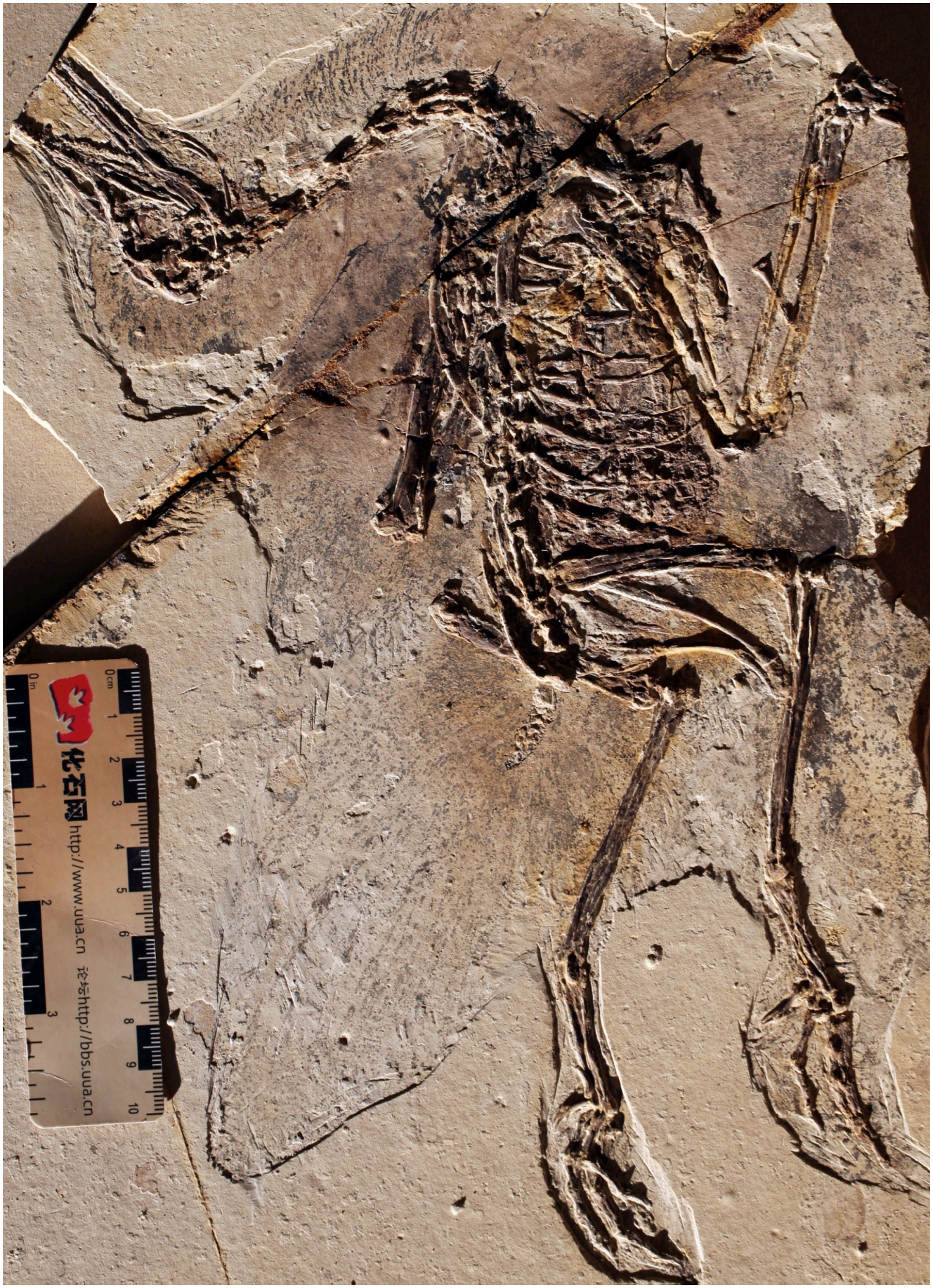

Figure S9. Photograph of *Yanornis* STM9-51 preserving a few isolated gastroliths in the ventriculus.

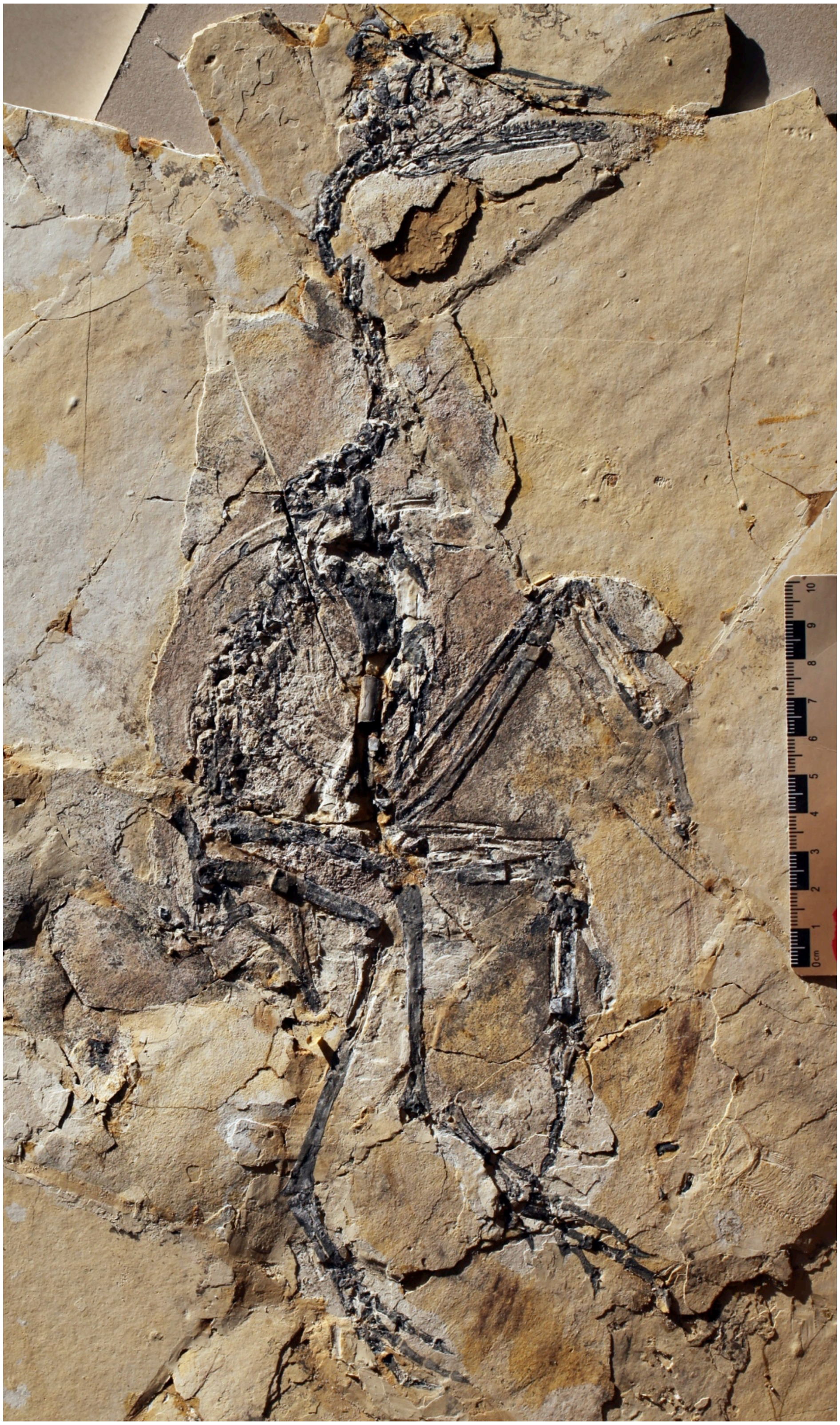

Figure S10. Photograph of *Yanornis* STM9-52

Cladistic Analysis

We used a modified version of the O'Connor and Zhou (2012) Mesozoic bird dataset. Two taxa now known to be non-avian theropods were removed (e.g. *Rahonavis*, *Zhongornis*). We added two new characters related to the alimentary canal:

246. Oesophageal crop: absent (0); simple swelling (1); pouch-like (2); distinct, lobe-shaped, distally located (3). [In piscivorous neornithines the crop is a simple swelling (1); in birds of prey the crop is pouch-like (2); in gallinaceous birds the crop is lobe-shaped (3); the crop is absent in owls (0).]

247. Grinding gizzard, as indicated by the use of true gizzard stones/gastroliths: absent (0); present (1). [A grinding gizzard is absent in birds of prey (0), but present in gallinaceous birds (1).]

The analysis was run using TnT (Goloboff et al., 2008). We preformed a heuristic search using tree-bisection reconnection (TBR) retaining the shortest tree out of a thousand producing 13 trees of 796 steps. A second round of TBR produced 116 trees of the same length (796 steps). The strict consensus tree differs from that of other analyses in that Sapeornithiformes is resolved as more closely related to Ornithothoraces (Confuciusornithiformes resolved in a more basal position) and *Jianchangornis* and *Archaeorhynchus* are basal ornithothoracines (usually resolved as basal ornithuromorphs). A crop is resolved as a synapomorphy of *Jeholornis* + Pygostylia. A ventrally located crop (246: 3) is a synapomorphy of Ornithothoraces. A grinding gizzard (247: 1) is resolved as a synapomorphy of Sapeornithiformes + Ornithothoraces, and secondarily lost in Enantiornithes.

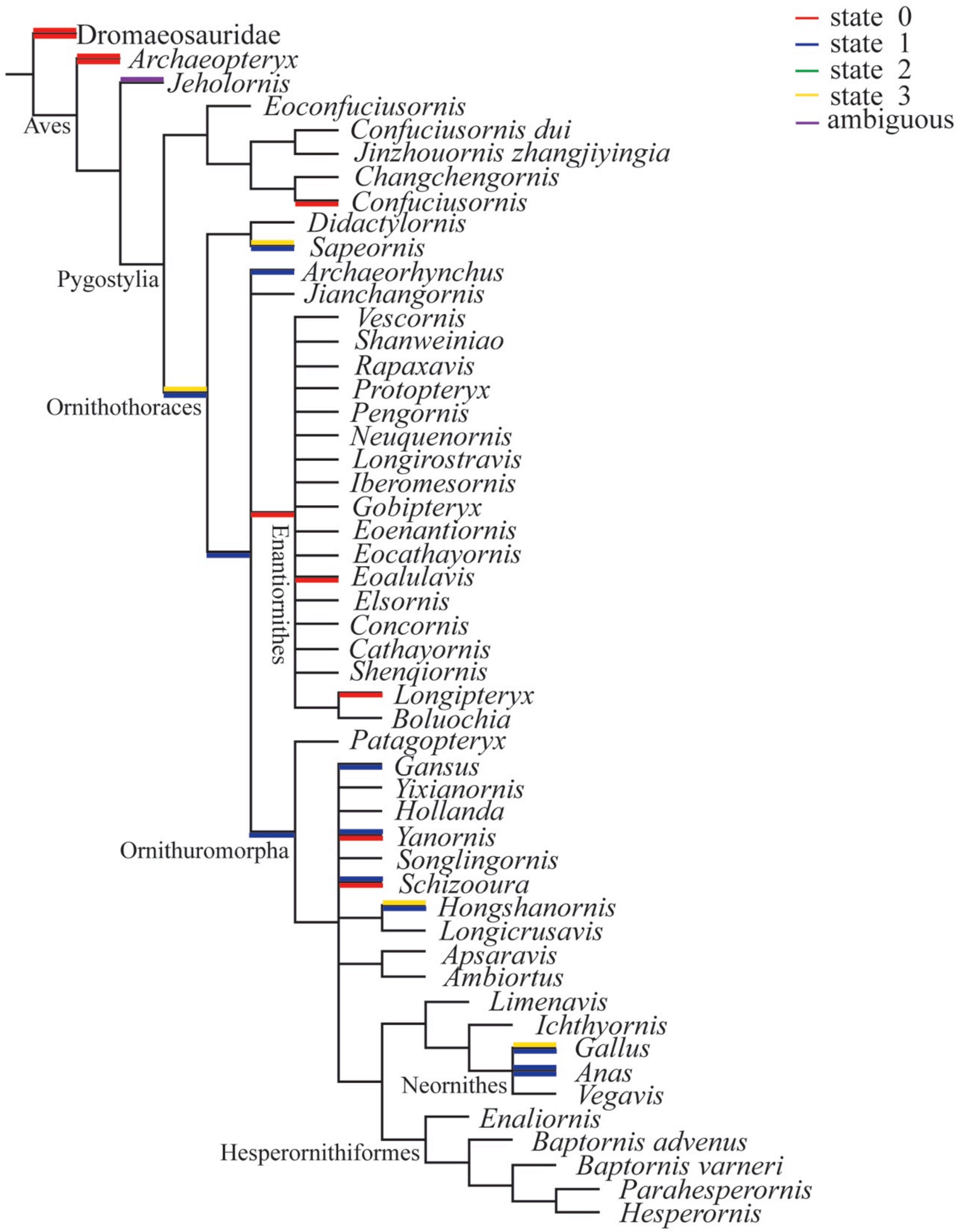

Figure S11. Strict consensus tree of 116 trees (L = 796 steps) with alimentary canal characters (characters 246, 247) mapped across. Scorings for character 246 are marked above the branch and 247 are mapped below.
